# The Crystal Structure and Intermolecular Interactions in Fenamic Acids–Acridine Complexes

**DOI:** 10.3390/molecules26102956

**Published:** 2021-05-16

**Authors:** Marta S. Krawczyk, Adam Sroka, Irena Majerz

**Affiliations:** Faculty of Pharmacy, Wrocław Medical University, Borowska 211a, 50-556 Wrocław, Poland; adam.sroka@umed.wroc.pl (A.S.); irena.majerz@umed.wroc.pl (I.M.)

**Keywords:** fenamic acids, acridine, complex, QTAIM, NCI approach

## Abstract

In order to improve pharmaceutical properties of drugs, complexes are synthesized as combinations with other chemical substances. The complexes of fenamic acid and its derivatives, such as mefenamic-, tolfenamic- and flufenamic acid, with acridine were obtained and the X-ray structures were discussed. Formation of the crystals is determined by the presence of the intermolecular O–H^…^N hydrogen bond that occur between fenamic acids and acridine. Intermolecular interactions stabilizing the crystals such as π^…^π stacking, C–H^…^X (X = O, Cl) intermolecular hydrogen bonds as well as C–H^…^π and other dispersive interactions were analyzed by theoretical methods: the quantum theory of atoms in molecules (QTAIM) and noncovalent interaction (NCI) approaches.

## 1. Introduction

N-arylanthranilic acids (fenamic acids) are popular drugs which belong to non-steroidal anti-inflammatory drugs (NSAIDs). The main compound of this group—fenamic acid—is not used in therapy because of side effects and low water solubility but fenamic acid derivatives, such as mefenamic-, tolfenamic-, flufenamic- and meclofenamic acid, are popular drugs widely used for the treatment of inflammation, pain, fever and tumor [1,2]. To improve bioavailability, solubility and other physicochemical properties important in pharmacy, fenamic acids can be used as salts with metal cations or molecular complexes with other organic compounds. The first step of investigation of physicochemical properties is characterization of the crystal structure of the drug compound—the description of the packing of the molecules in a crystal [3] which is a result of intermolecular interactions [4].

**Figure 1:**
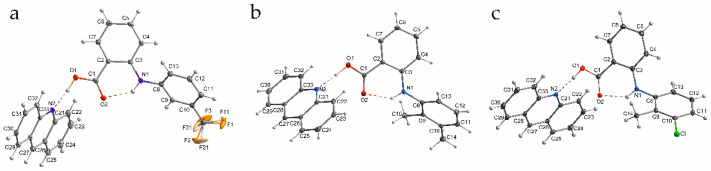
The investigated fenamic acid–acridine complexes: (**a**) flufenamic (**1**), (**b**) mefenamic (**2**) and (**c**) tolfenamic acid (**3**).

As the prototype of fenamic acid complexes with tertiary amines, the complex of fenamic acid with acridine was investigated previously [5]. The results presented in this work are a continuation of the structure analysis of acridine complexes with fenamic acids used in therapy: flufenamic, mefenamic and tolfenamic. The structures of the investigated complexes are presented in Figure 1.

The intermolecular interaction between the molecules in the crystal have been described using the Quantum Theory of Atom in Molecules (QTAIM) [6] and the Noncovalent Interaction description (NCI) [7,8]. The QTAIM is a theoretical method commonly used to investigate the covalent and hydrogen bond as well as weak noncovalent interaction [9,10]. The main evidence of the bond or interatomic interaction is the presence of a bond path with a bond critical point (BCP) linking two atoms and the electron density at the BCP (ρ(r)) is related to the strength of the interaction. The sign of Laplacian of the electron density at BCP differentiates the interaction type. Negative Laplacian typical for covalent and polarized bonds indicates so called “shared interaction” connected with concentration of electron density between interacting atoms. Positive sign of the Laplacian is typical for hydrogen bonds, van der Waals and ionic interactions and indicates depletion of the electron density between the interacting atoms. For a noncovalent (“closed-shell”) interaction, the value of the electron density at BCP has to be in the 0.024–0.139 au range [10]. For weak interaction, except the electron density at the BCP, also other QTAIM parameters have to be taken into account: the ellipticity of the electron density (ε) cannot be very high and the bond path linking interacting atoms cannot be very bent [10]. 

In the frame of QTAIM theory also the energetic properties of electron density at the BCP can be analyzed—potential energy density, V(r), and electronic kinetic energy, G(r)—at a BCP. The potential energy density of electrons at BCP (V(r)) expresses the pressure exerted on the electrons by the other electrons. The kinetic energy (G(r)) is connected with mobility of the electron density at the BCP and reflects the pressure exerted by the electrons at BCP on the other electrons [11,12]. Concerning the chemical bonding studies in crystals of complexes, a recently developed method called “periodic local vibrational mode theory” is also a tool that may be used in analysis of interactions [13]. 

To characterize more precisely very weak interactions, the Noncovalent Interaction (NCI) approach can be applied [7]. This method uses two types of visualization of very weak hydrogen bonds, van der Waals interactions, dispersion and steric repulsion—the NCI plot and NCI surface. The NCI plot is a function of the reduced density gradient versus the electron density multiplied by the sign of the second Hessian of electron density eigenvalue (λ_2_). The spikes located at the negative values indicate the attractive noncovalent interactions. The spikes at the positive values correspond to the steric repulsion. Two spikes symmetrically located around the zero value in the NCI plot indicate the presence of the dispersive interaction. The second visualization method of noncovalent attractive interaction and steric repulsion used in the frame of NCI approach is the gradient iso-surfaces in real space for the molecule. The traditionally used color for the iso-surfaces is green for attractive, and red for the repulsive interaction. 

## 2. Results

### 2.1. The Inter- and Intramolecular Hydrogen Bonds of Fenamic Acids–Acridine Complexes 

The main interaction linking acridine and fenamic acids molecules in the crystal structures is the intermolecular OHN hydrogen bond in which the proton of the carboxyl group is involved in formation of a hydrogen bond with the acridine nitrogen atom. The crystal data are given in Table 1. Fenamic acid–acridine complex is a typical benzoic acid—a tertiary amine cocrystal with an OHN hydrogen bond. Geometrical parameters of the OHN hydrogen bonds of the studied complexes along with acridine–fenamic acid complex investigated previously [5] are presented in Table 2. 

The O–H and N^…^H bond lengths confirm the molecular character of the OHN hydrogen bond. The O–H bond is elongated comparing the bond length to the fenamic acid but the proton is still located at the donor oxygen atom and the N^…^H distance is significantly longer than the O–H. The O^…^N hydrogen bond length is typical for the weak OHN hydrogen bond. Except changes of the bond lengths in the hydrogen bond bridges, the molecular character of the OHN hydrogen bond is also reflected in the C–O and C=O bond lengths of the carboxylic group. For the investigated complexes, both CO bonds are different in comparison to the strongest hydrogen bond with the central location of the proton between donor and acceptor where the CO bond lengths of the carboxyl group are similar [14]. In addition to the main intermolecular OHN hydrogen bond linking the fenamic acid with acridine, the intramolecular NHO hydrogen bond linking the NH group of fenamic acid with the oxygen atom of the carbonyl group is also present. This intramolecular hydrogen bond is typical for fenamic acids [15,16,17,18,19] and also occurs in the fenamic acid complexes. 

The geometrical parameter which is not connected to the hydrogen bonds but is sensitive to the packing of the molecules in crystal is the angle between the fenamic acid aromatic planes. It is 44.92(5)° for the complex of fenamic acid with acridine, 51.37(5)° for the complex of flufenamic acid, 62.71(4)° for the complex of mefenamic acid (**2**) and 61.68(4)° for the complex of tolfenamic acid with acridine (**3**). 

### 2.2. The Packing of the Molecules in Crystals 

**Figure 2:**
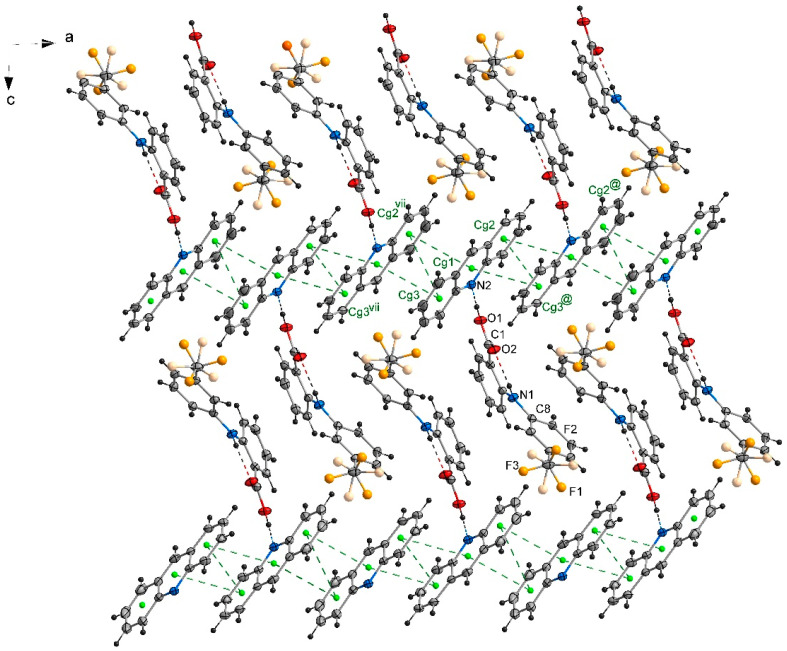
Packing diagram of crystal 1 viewed down the [010] direction showing arrangement of the complexes of acridine and flufenamic acid. Stacking interactions of acridine molecules are shown by green dashed lines linking centers of aromatic rings (centroids of rings-Cg-marked as green balls; Cg1 (N2/C21/C26/C27/C28/C33), Cg2 (C21/C22/C23/C24/C25/C26), Cg3 (C28/C29/C30/C31/C32/C33)). Symmetry codes: @ = −x, −y + 2, −z; vii = −x + 1, −y + 2, −z.

**Figure 3:**
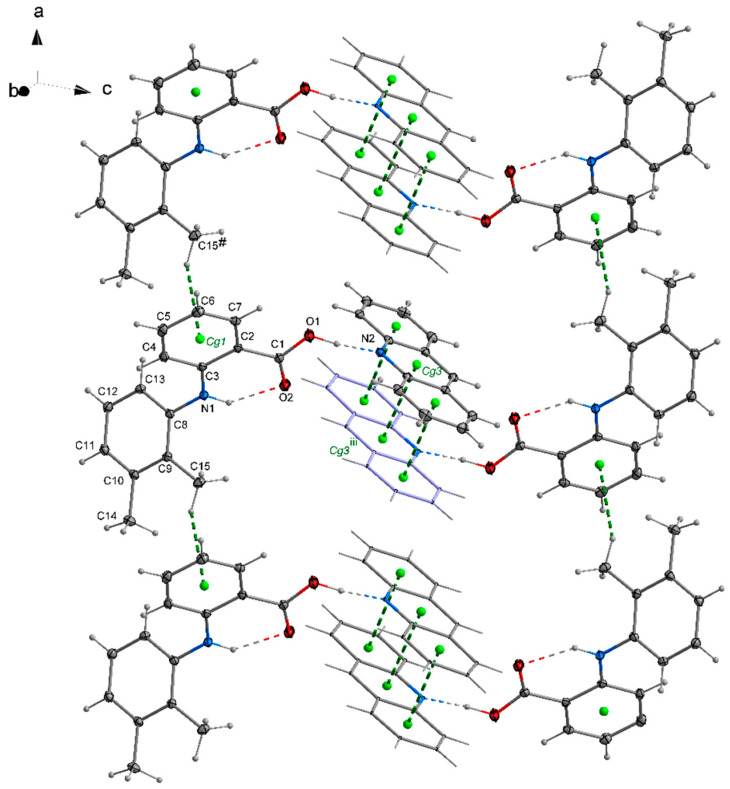
Arrangement of mefenamic acid and acridine molecules in **2** forming a double chain stretching along the [100] direction. Stacking of acridine molecules and C–H^…^π interactions between mefenamic acid molecules (Table S1, Supplementary Materials) are shown by green dashed lines linking centers of aromatic rings (centroids of rings-Cg-marked as green balls; Cg1 (C2/C3/C4/C5/C6/C7), Cg3 (N2/C21/C26/C27/C28/C33)) Symmetry codes: # = 1 + x, y, z; iii = −x + 1, −y, −z + 1.

**Figure 4:**
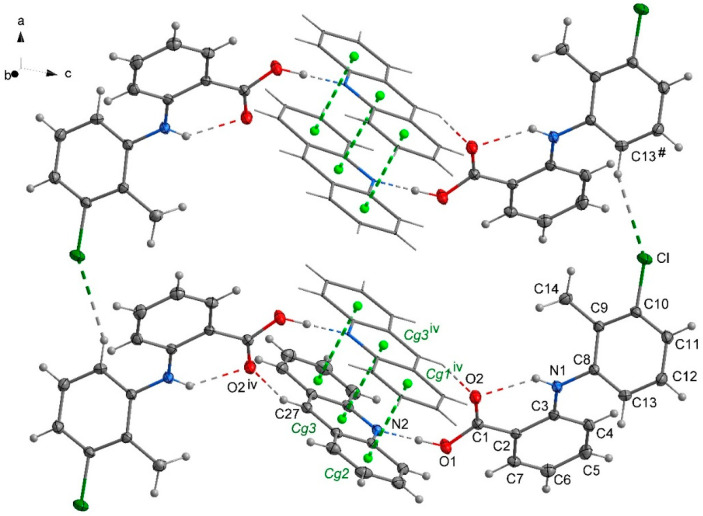
Packing crystal **3** showing arrangement of the complexes of acridine and tolfenamic acid. Stacking interactions of acridine molecules are shown by green dashed lines linking centers of aromatic rings (centroids of rings-Cg-marked as green balls; Cg1 (C28/C29/C30/C31/C32/C33); Cg2 (C21/C22/C23/C24/C25/C26); Cg3 (N2/C21/C26/C27/C28/C33)). Symmetry codes: iv = −x + 1, −y + 1, −z; # = x + 1, y, z.

In the crystal **1**, a chain motif extending along the [100] direction and composed of acridine molecules participating in stacking interactions is observed (Figure 2). The “branches” of the chain are formed by flufenamic acid molecules interacting with the acridine chain via O1–H1O^…^N2 hydrogen bonds. Flufenamic acid molecules that belong to the adjacent chains located in the crystallographic plane (010), are related by the 2-fold screw axis (½-x, ½+y, ½-z). The crystal of **2** reveals a double chain motif resembling “rungs of a ladder” extending towards the [100] direction (Figure 3). Within each of the chains, the molecules of mefenamic acid contact each other via C–H^…^π interactions (C15–H15b^…^Cg1^#^, # = x + 1, y, z) (Table S1, Supplementary Materials) while two adjacent chains are linked by π^…^π interactions of acridine rings forming “rungs of a ladder”. The analogous arrangement of complex components is observed in the crystal of tolfenamic acid with acridine (**3**) (Figure 4). Double chains stretching along the [100] direction are created by molecules of tolfenamic acid contacting each other via C13–H13^…^Cl^ix^ (d(D^…^A) = 3.6977(16) Å; <(D–H^…^A) = 153°; ix = x–1, y, z), resulting in formation of chains between which the π^…^π stacked acridine molecules occur.

### 2.3. General Motifs in the Crystals of Acridine–Fenamic Acids 

**Figure 5:**
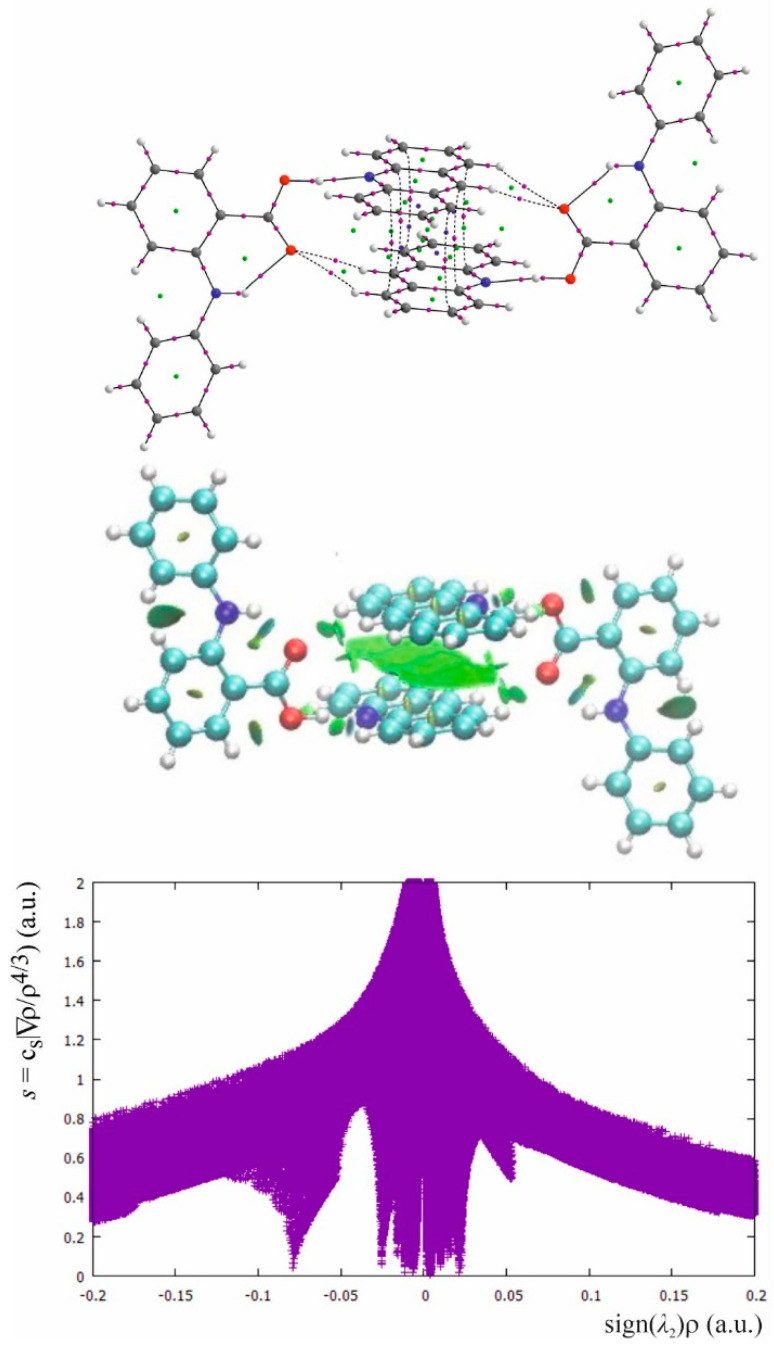
The QTAIM and NCI diagram, and NCI plot for fenamic acid–acridine complex common also for the complexes of mefenamic and tolfenamic acid.

The main intermolecular interaction which determines the packing of the molecules in the crystals of fenamic acids–acridine complexes is the OHN hydrogen bond linking the fenamic acid with acridine. Except the OH which is a proton donor, the carboxylic group contains the C=O oxygen atom which can act as an acceptor of the proton. The packing of the molecules in the investigated crystals is determined by the ability of these oxygen atoms to be a proton donor and a proton acceptor. In studied crystals of the complexes of acridine with fenamic, mefenamic (**2**) and tolfenamic acid (**3**), the hydrogen-bonded complexes of the fenamic acid with acridine are organized in dimers joined by two OHN hydrogen bonds and the CHO hydrogen bonds formed by C27–H27 linked to the O2 oxygen atom of the C1=O2 group. In Figure 5, QTAIM and NCI diagram for acridine–fenamic acid are presented. In Table 3, the QTAIM parameters for the bond critical points, which illustrates the strength of the hydrogen bonds, are collected.

In the OHN hydrogen bond linking the fenamic acid with acridine, the OH bond is typically a covalent bond with a negative value of Laplacian of the electron density at BCP. The H^…^N is a closed shell interaction with significantly lower electron density than for the OH bond and with a positive Laplacian. The electron density and the sign of the Laplacian of the electron density at BCP for OH and H^…^N confirms molecular character of the OHN hydrogen bond which can be classified as rather weak. Comparing the parameters in Table 3, it is evident that the CHO interactions between acridine and fenamic acid are distinctly weaker than the main OHN hydrogen bond. According to Popelier criteria for weak hydrogen bonds [10], except the value of the electron density at BCP, also the ellipticity and nonlinearity of the bond path should be taken into account and the interaction with very high ellipticity and nonlinearity can vanish. The CH^…^O interactions are linear but for mefenamic- and tolfenamic acid the CHO with the longer H^…^O distance is characterized by high ellipticity that expresses instability of this interaction. Green surfaces in NCI diagrams confirm weakness of the OHN hydrogen bond as well as dispersive character of the CHO interactions. The NCI plot is rather complex. The OHN and NHO hydrogen bonds are represented by a broad, intensive spike at about −0.08 of the electron density multiplied by the sign of the second eigenvalue of the Hessian of the electron density at BCP. The low intensity spike at about 0.05 is connected with repulsive interactions in the centers of the aromatic rings. A very broad group of spikes located symmetrically around zero is connected with many dispersive interactions for which the spikes symmetrically located around zero are typical.

**Figure 6:**
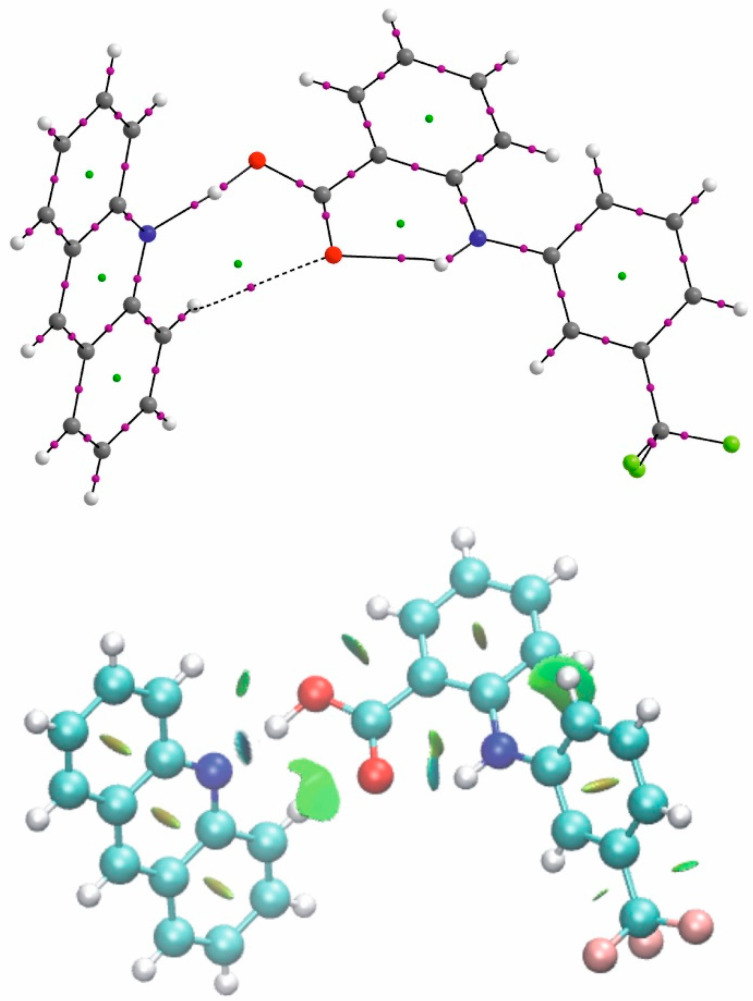
The QTAIM and NCI diagram for flufenamic acid–acridine complex.

In the case of the crystal of flufenamic acid with acridine (**1**), the ability of the C=O oxygen to accept a proton is expressed by formation of the CHO interaction between the oxygen and the proton of the acridine molecule (*viz*. C22–H22^…^O2) of the same acridine which is engaged in the OHN hydrogen bond. This interaction is very weak (d(C22–O2) = 3.395(3) Å; d(H22–O2) = 2.86 Å) which is expressed by high ellipticity of the H^…^O electron density at BCP and additionally the bond path is nonlinear. The NCI diagram differentiates the intermolecular OH^…^N and intramolecular NH^…^O hydrogen bond to the CH^…^O interaction and the green surface around oxygen expresses its dispersive character (Figure 6).

### 2.4. Stacking Interactions in Acridine–Fenamic Acids Complexes 

Stacking is a very common interaction in the solid state, liquids and in the gas phase which is responsible for mutual organization of the aromatic molecules. When the distance between the aromatic rings is lower than the van der Waals radii (3.5 Å), interaction of the aromatic electron clouds is possible. Parallel arrangement of the aromatic molecules is the best for the interaction of the π aromatic electrons, but the stacking interaction between the aromatic rings can also be realized as a parallel displaced, edge-to-face or T-shaped arrangement of the aromatic molecules [20,21,22]. To investigate the stacking interaction, geometry of the interacting aromatic rings should be taken into account. The geometrical parameters which describe arrangement of the aromatic rings are: stacking distance *h*—the distance between the center of one ring and a plane of another ring, *shift r* (*displacement*)—the distance between the two centers projected on a plane defined by atoms of one ring, inclination *θ* (*tilt*) of one ring plane to the other ring plane, twist *φ* of one ring in relation to another, direction *ψ* of the shift *r* in relation to the chosen, specific direction [23]. 

The stacking interaction between the aromatic acridine rings is present for every investigated complex of acridine with fenamic acid and for the complex of mefenamic- and tolfenamic acid two different stacking complexes are present. Geometrical and QTAIM stacking parameters for the acridine rings of the investigated fenamic acid–acridine complexes are collected in Table 4. For every acridine–fenamic acid complex, the distance between the acridine planes is less than 3.5 Å so the stacking interaction is possible. All stacking rings are parallel displaced and the distance between the two centers projected on a plane defined by atoms of one ring shows that the center of one ring is located under the CC bond of the second interacting ring. The angles between the stacked acridine ring planes are close to zero, similarly to other acridine–carboxylic acids complexes [24].

**Figure 7:**
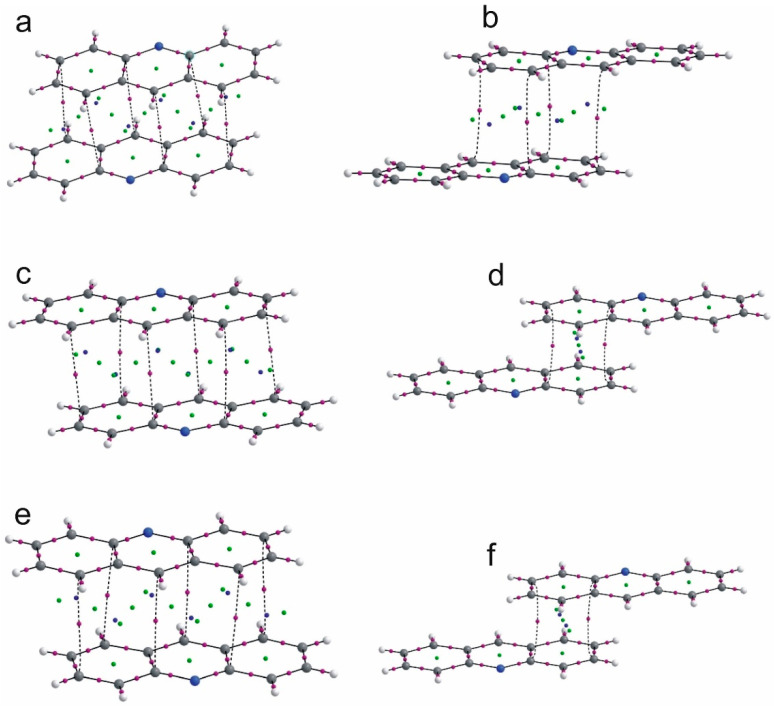
QTAIM diagrams for (**a**)—acridine–fenamic acid, (**b**)—acridine–flufenamic acid (**1**), (**c**,**d**)—acridine–mefenaic acid (**2**_stack1, **2**_stack2, Table 4) and acridine–tolfenamic acid ((**e**,**f**)—**3**_stack1, **3**_stack2, Table 4).

Stacking interactions between the acridine aromatic rings are expressed by the bond paths linking the carbon atoms of the interacting rings. In Figure 7, the QTAIM diagrams for the interacting acridine rings for the investigated complexes are presented.

There are three types of the rings arrangements: the parallel acridine ring planes linked with six bond paths, two acridine ring planes shifted so only two bond paths between one acridine ring can be formed and the case of fenamic acid–acridine complex with four bond paths linking two acridine rings. For every arrangement of the acridine rings, the electron density, kinetic and potential energy of the electron density at C^…^C BCP confirm that the interaction is very weak, weaker than in the case of weak CH^…^O hydrogen bond. For flufenamic acid–acridine complex, the ellipticity at BCP for the C^…^C interaction between the acridine rings is higher than for other investigated complexes. For the stacking interaction of one acridine ring with two C^…^C bond paths, the bond paths are more nonlinear than in the case of other stacking interactions. 

**Figure 8:**
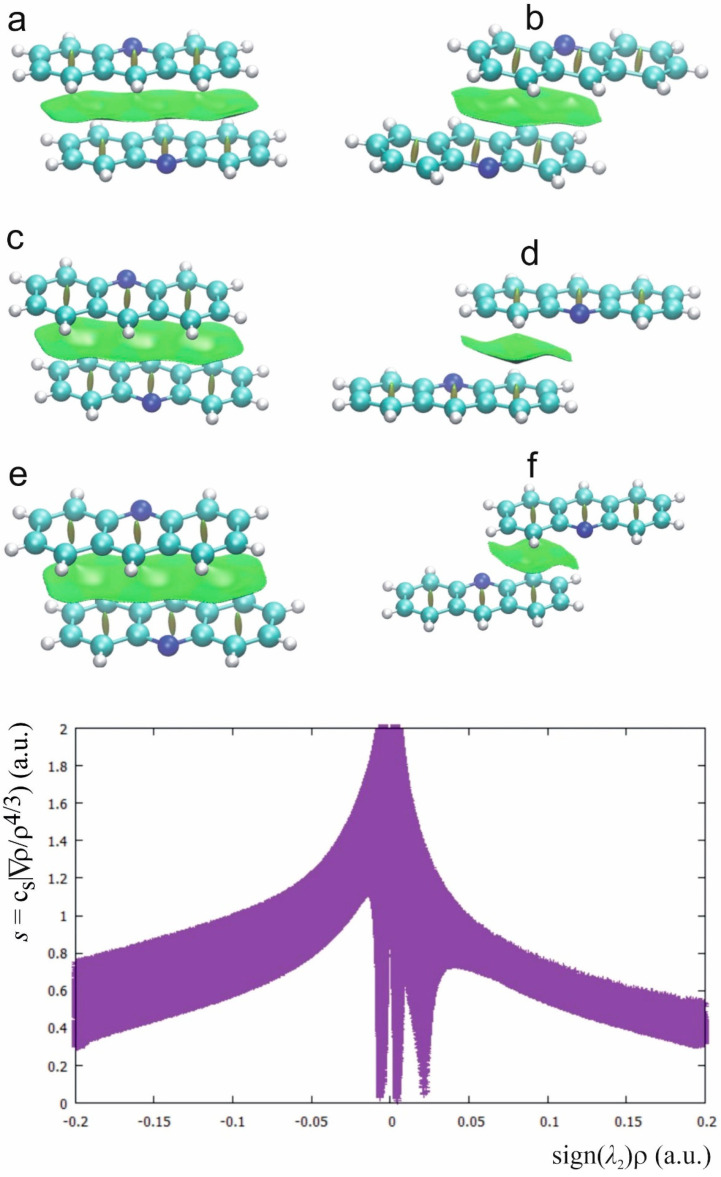
NCI diagrams for (**a**) acridine–fenamic acid, (**b**) acridine–flufenamic acid (**1**), (**c**,**d**) acridine–mefenaic acid (**2**_stack1, **2**_stack2, Table 4) and acridine–tolfenamic acid ((**e**,**f**) **3**_stack1, **3**_stack2, Table 4).

The NCI plot for the investigated complexes (Figure 8) confirms the nature of the interaction between the acridine rings. The green surfaces between the acridine rings indicate that the interaction has dispersive character. The NCI plot in Figure 8 common for all investigated complexes contains the spikes symmetrically located around zero which are typical for dispersion and the spikes connected with repulsive interaction in the center of the acridine rings. Comparison of the NCI plot in Figure 5 and Figure 8 allows to find the spikes connected with dispersive stacking interactions between the acridine rings and other weak interactions as CH^…^O hydrogen bond. 

To interpret the bonding force linking the acridine molecules in the investigated complexes of fenamic acids with acridine, the energy decomposition according to Morokuma–Ziegler has been performed [25,26]. The bonding energy is decomposed into electrostatic (E_elect_), Pauli (E_Pauli_), orbital (E_orb_) and dispersive (E_dysp_) components (Table 5).

E_bonding_ = E_elect_ + E_Pauli_ + E_orb_ +E_dysp_

E_elect_ is the Coulomb interaction between the unperturbed charge of acridine molecules. E_Pauli_ expresses the destabilizing Pauli repulsion, E_orb_ indicates the interaction energy between the orbitals of two acridine molecules, E_dysp_ is the dispersion energy for the intermolecular van der Waals interaction between two acridine molecules. The sum of E_elect_ and E_Pauli_ is the steric interaction.

It is characteristic that the total energy is the highest when the interaction contains three aromatic rings of acridine. For flufenamic acid–acridine complex, with two acridine rings engaged in the stacking interaction the energy is lower and for the stacking with only one acridine ring engaged in the stacking it is the lowest. The main component of the energy interaction between the acridine rings is the dispersive interaction and the energy values in Table 5 show that the dispersion is responsible for the stacking of the acridine molecules in the complexes of acridine with fenamic acids. Despite the short distance between the acridine planes, the orbital interaction is very low. The π^…^π electron interaction has repulsive character and the energy decomposition in Table 5 confirms that it is related to the area of the π-electrons overlap. To diminish the repulsion, the aromatic rings of the stacking acridines are shifted so the center of the aromatic ring is located over the C-C bond of the other interacting ring. 

**Figure 9:**
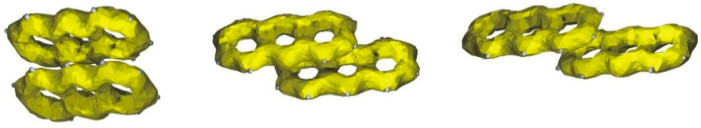
ACID surfaces for different arrangements of the stacking acridine molecules.

Aromatic molecules are characterized by delocalization of the electron density in the aromatic ring, which is related to reactivity and many physical and chemical properties. A method that can be interpreted as a representation of delocalized electrons and visualize the electron mobility is Anisotropy of the Current-Induced Density (ACID) [27]. It can be expected that the stacking interaction should influence the delocalization of the electrons in acridine aromatic rings. According to Figure 9, regardless of the π-electron surface involved in the stacking interaction, delocalization of the electrons is not sensitive to the stacking and the aromatic character of the rings is dominant, hence the weak interaction between the aromatic rings does not influence the delocalization and mobility of the electrons.

### 2.5. Additional Weak Interactions in the Crystal Structure of the Investigated Acridine Complexes

**Figure 10:**
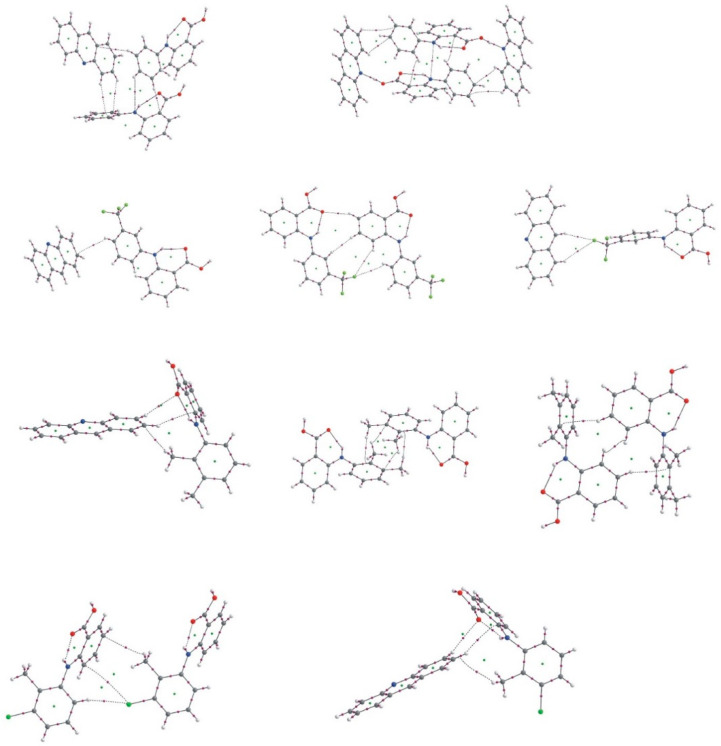
QTAIM diagrams for weak intermolecular interactions in the fenamic acids–acridine complexes.

When the strong inter- and intramolecular hydrogen bonds and stacking interactions are common for the complex of acridine with fenamic-, flufenamic, mefenamic- and tolfenamic acid, other weak interactions, in particular crystal, arise because of the specific substituent in the acid ring or special packing of the molecules in the crystal. In Figure 10 the QTAIM diagrams for these interactions are presented. 

The CF_3_ group in flufenamic acid and Cl atom in tolfenamic acid can be very weak proton acceptors. The methyl group in tolfenamic- and mefenamic acid forms a very weak interaction with the aromatic ring marked as a bond path linking the methyl hydrogen with the carbon atom of the aromatic ring. The hydrogen atoms of aromatic rings of fenamic acid also can form very weak CH^…^O, CH^…^C or CH^…^π hydrogen bonds. The interactions mentioned above are very weak so in the NCI diagrams they are marked as the green surfaces. More of them, such as CH_3_^…^π, are very bent and the ellipticity of the electron density at BCP is high which indicates instability of these interactions.

## 3. Materials and Methods

### 3.1. Crystal Preparation

All reagents and solvents were used as obtained commercially without further purification. Mefenamic, tolfenamic and flufenamic acid were purchased from Sigma-Aldrich Sp.z.o.o Poznan, Poland. Solvents: dimethyl sulfoxide (DMSO) and ethanol were purchased from Avantor Performance Materials Poland S.A.

In the case of **1** and **3** complexes, mixtures of acridine with tolfenamic and flufenamic acids respectively in ratios 1:1 were placed in flasks and refluxed for several minutes in ethanol. Subsequently, after cooling, mixtures were poured into crystallizers, covered by parafilm and left for crystallization under normal pressure. Crystals of the complex **1** were obtained by dissolving the mefenamic acid and acridine in the ratio 1:1 in dimethyl sulfoxide and left for slow evaporation under atmospheric pressure. 

### 3.2. X-ray Diffraction

Crystals of fenamic acid–acridine complexes were collected on a KUMA KM4 and Xcalibur Ruby four-circle diffractometers equipped with CCD area detectors using Mo *K*α (λ = 0.71073 Å) radiation. 

Structures were solved by direct methods and refined by the full-matrix least-squares method using *SHELXL2014* software [28,29]. The crystal data and structure refinements for the investigated compounds are presented in Table 1. *DIAMOND* [30] program was used for molecular graphics. 

All hydrogen atoms in structures **1**–**3** were found in the difference Fourier map. The hydrogen atom from carboxyl group was refined freely with an isotropic displacement parameter and positions of hydrogen atoms from aromatic rings and methyl groups (in **2** and **3**), were treated as riding atoms in geometrically idealized positions, with C—H distance restrained to 0.95 Å, *U*_iso_(H) = 1.2*U*_eq_(C) for aromatic rings and C—H distance restrained to 0.98 Å, *U*_iso_(H) = 1.5*U*_eq_(C) for methyl groups in **2** and **3**.

The crystallographic data have been assigned the following deposition CCDC Numbers: 2081569-2081571. 

### 3.3. Computational Details

The molecular structures obtained from the X-ray were used to generate the wave function with Gaussian 16 package [31] at DFT B3LYP/6-311++G** level [32,33] without further optimization. The wave function for the molecules in a crystal structure were taken as an input to the AIMALL [34] and to the NCI program [35].

The energy decomposition procedure according to Morokuma–Ziegler [25,26] was implemented to ADF suite ADF2019.03, SCM [36] was performed for the acridine molecules with the solid state geometry.

## 4. Conclusions

Strong O–H^…^N hydrogen bonds are mainly responsible for the arrangement of molecules in co-crystals of fenamic acids with acridine. The investigated crystals are stabilized by π^…^π stacking interactions, as well as by C–H^…^X (X = O, Cl) intermolecular hydrogen bonds, C–H^…^π and other dispersive interactions.

## Figures and Tables

**Table 1:** Crystallographic data and refinement parameters for fenamic acids–acridine complexes.

Crystal Data	1	2	3
Chemical formula	C_14_H_10_F_3_NO_2_ · C_13_H_9_N	C_15_H_15_NO_2_ · C_13_H_9_N	C_14_H_12_ClNO_2_ · C_13_H_9_N
*M_r_*	460.44	420.49	440.91
Crystal system, space group	Monoclinic, *P*2_1_/*n*	Triclinic, *P*-1	Triclinic, *P*-1
Temperature (K)	100(2)	100(2)	100(2)
*a* (Å)	9.348(3)	8.052(3)	8.0560(18)
*b* (Å)	7.976(2)	9.788(3)	9.6596(19)
*c* (Å)	29.100(9)	14.695(4)	14.880(3)
*α* (°)		103.10(3)	103.69(2)
*β* (°)	92.86(3)	101.01(3)	101.73 (2)
*γ* (°)		98.15(3)	99.44(2)
*V* (Å ^3^)	2167.0(11)	1086.3(6)	1073.8(4)
*Z*	4	2	2
Radiation type	Mo *K*α	Mo *K*α	Mo *K*α
Crystal size (mm)	0.37 × 0.30 × 0.28	0.70 × 0.67 x 0.60	0.18 × 0.18 × 0.15
Data collection			
Diffractometer	Xcalibur, Ruby, Gemini ultra-CCD κ-geometry diffractometer	Xcalibur, Ruby-CCD κ-geometry diffractometer	XtaLAB Synergy R, DW system, HyPix-Arc 150 diffractometer
No. of measured, independent and observed [I >2*σ*(*I*)] reflections	24291, 6404, 5076	9125, 5884, 4667	15700, 5380, 4656
*R_int_*	0.025	0.020	0.017
Refinement			
*R*[*F*^2^ > 2*σ*(*F*^2^)], *wR*(*F*^2^), *S*	0.047, 0.118, 1.03	0.047, 0.126, 1.04	0.040, 0.115, 1.04
No. of parameters	342	297	296
No. of restraints	0	0	0
H-atom treatment	H atoms treated by a mixture of independent and constrained refinement	H atoms treated by a mixture of independent and constrained refinement	H atoms treated by a mixture of independent and constrained refinement
Δρ_max_, Δρ_min_ (e Å^−3^)	0.37, −0.27	0.38, −0.25	1.18, −0.36

**Table 2:** Selected geometrical parameters of the investigated complexes of fenamic acids with acridine including inter- and intramolecular hydrogen bonds.

			O1–H1O^…^N2				N1–H1^…^O2			
Complex	C1-O1 [Å]	C1=O2 [Å]	O1–H1O [Å]	H1O^…^N2 [Å]	O1^…^N2 [Å]	OHN [°]	N1–H1 [Å]	H1^…^O2 [Å]	N1^…^ O2 [Å]	NHO [°]
acridine–fenamic acid [5]	1.3162(18)	1.2336(18)	1.06(2)	1.55(2)	2.609(2)	173(2)	0.886(16)	1.99(2)	2.691(2)	135(2)
Acridine–flufenamic acid (**1**)	1.3221(14)	1.2292(15)	0.990(18)	1.626(18)	2.6118(14)	173.2(16)	0.872(16)	1.958(15)	2.6714(15)	138.0(14)
Acridine–mefenamic acid (**2**)	1.3172(15)	1.2317(16)	1.021(19)	1.597(19)	2.6163(16)	175.0(16)	0.876(17)	1.944(16)	2.6660(16)	138.8(14)
Acridine–tolfenamic acid (**3**)	1.3153(16)	1.2322(16)	0.89(2)	1.72(2)	2.6103(16)	175(2)	0.812(19)	2.011(19)	2.6692(16)	137.8(17)

**Table 3:** QTAIM parameters for the BCPs. Symmetry codes: i = −x + 1, −y + 1, −z + 2; ii = x + 1, y, z + 1; iii = −x + 1, −y, −z + 1, iv = −x + 1, −y + 1, −z.

Interaction	Bond Length/Interatomic Distance [Å]	ρ(r)	∇^2^(r)	V(r)	G(r)	ε(r)	d [Å]
Fenamic acid^…^acridine							
O1–H1O	1.0617	0.2637	−1.4900	−0.5182	0.0728	0.0148	0.0006
H1O∙∙∙N2 ^i^	1.5508	0.0789	0.0795	−0.0792	0.0495	0.0291	0.0004
O2∙∙∙H27A ^ii^	2.4505	0.0088	0.0315	−0.0051	0.0065	0.0542	0.0127
O2∙∙∙H25A ^ii^	2.7734	0.0048	0.0170	−0.0028	0.0035	0.0884	0.0229
**1**							
O1–H1O	0.990(18)	0.2659	−1.5189	−0.5191	0.0697	0.0156	0.0006
H1O∙∙∙N2	1.626(18)	0.0731	0.0857	−0.0718	0.0466	0.0337	0.0007
O2∙∙∙H22	2.860	0.0043	0.0162	−0.0025	0.0033	0.2766	0.1311
**2**							
O1–H1O	1.021(19)	0.2981	−1.9274	−0.6299	0.0740	0.0144	0.0004
H1O∙∙∙N2	1.597(19)	0.0699	0.0992	−0.0697	0.0472	0.0288	0.0002
O2∙∙∙H27 ^iii^	2.4288	0.0089	0.0328	−0.0052	0.0067	0.0531	0.0132
H25∙∙∙O2 ^iii^	3.0584	0.0028	0.0108	−0.0015	0.0021	0.3773	0.0381
**3**							
O1∙∙∙H1O	0.89(2)	0.2705	−1.5980	−0.5406	0.0706	0.0147	0.0007
N2∙∙∙H1O	1.72(2)	0.0727	0.0851	−0.0712	0.0462	0.0295	0.0001
O2∙∙∙H27 ^iv^	2.441	0.0068	0.0243	−0.0040	0.0050	0.0529	0.0160
O2∙∙∙H25 ^iv^	3.098	0.0024	0.0094	−0.0013	0.0018	0.3232	0.0388

Symbols: ρ(r)—electron density at the BCP (bond critical point); ∇^2^(r)—Laplacian electron density; V(r)—potential energy density; G(r)—kinetic energy; ε(r)—ellipticity of the electron density; d [Å]—the difference between the length of the bond path and the distance between the atoms linked by this bond.

**Table 4:** Geometrical and QTAIM stacking parameters for acridine rings in fenamic acids–acridine complexes. Symmetry codes: iii = −x + 1, −y, −z + 1; iv = −x + 1, −y + 1, −z; v = −x, −y + 1, −z; vi = −x + 2, −y, −z + 1; vii = −x + 1, −y + 2, −z; viii = −x, −y + 1, −z + 1.

Interaction	r [Å]		CC Distance with Bond Path	ρ(r)	∇^2^(r)	∇(r)	G(r)	ε(r)	d [Å]
Fenamic acid^…^acridine									
3.3157	1.4503	C23–C29 ^viii^	3.3128	0.0066	0.0180	−0.0029	0.0037	0.8757	0.0555
		C25–C33 ^viii^	3.3167	0.0063	0.0179	−0.0028	0.0037	0.2187	0.0343
		C21–C27 ^viii^	3.3209	0.0061	0.0177	−0.0028	0.0036	0.0386	0.0287
		C27–C21 ^viii^	3.3209	0.0061	0.0177	−0.0028	0.0036	0.0387	0.0287
		C33–C25 ^viii^	3.3167	0.0063	0.0179	−0.0028	0.0037	0.2188	0.0344
		C29–C23 ^viii^	3.3128	0.0066	0.0180	−0.0029	0.0037	0.8760	0.0555
**1**									
3.3968	1.3990	C27–C31 ^vii^	3.4323	0.0056	0.0144	−0.0024	0.0030	0.9382	0.0640
		C29–C33 ^vii^	3.3838	0.0056	0.0162	−0.0025	0.0033	0.9147	0.0459
		C33–C29 ^vii^	3.3838	0.0056	0.0162	−0.0025	0.0033	0.9147	0.0459
		C31–C27 ^vii^	3.4323	0.0056	0.0144	−0.0024	0.0030	0.9382	0.0640
**2**_stack1				0.0089	0.0328	−0.0052	0.0067	0.0531	0.0132
3.3645	1.4652	C23–C29 ^iii^	3.3719	0.0060	0.0161	−0.0026	0.0033	0.9936	0.0579
		C25–C33 ^iii^	3.3931	0.0056	0.0156	−0.0025	0.0032	0.3451	0.0396
		C21–C27 ^iii^	3.3661	0.0057	0.0162	−0.0025	0.0033	0.1225	0.0345
		C27–C21 ^iii^	3.3661	0.0057	0.0162	−0.0025	0.0033	0.1225	0.0345
		C33–C25 ^iii^	3.3931	0.0056	0.0156	−0.0025	0.0032	0.3451	0.0396
		C29–C23 ^iii^	3.3719	0.0060	0.0161	−0.0026	0.0033	0.9936	0.0579
**2**_stack2									
3.2858	1.7052	C21–C23 ^vi^	3.301	0.0066	0.0189	−0.0029	0.0038	0.6102	0.1315
		C23–C21 ^vi^	3.301	0.0066	0.0189	−0.0029	0.0038	0.6102	0.1315
**3**_stack1									
3.3645	1.4652	C21–C23 ^v^	3.3422	0.0060	0.0173	−0.0027	0.0035	0.4528	0.1053
		C23–C21 ^v^	3.3422	0.0060	0.0173	−0.0027	0.0035	0.4528	0.1053
**3**_stack2									
3.4202	1.3813	C23–C29 ^iv^	3.4052	0.0057	0.0149	−0.0024	0.0031	0.8305	0.0300
		C33–C25 ^iv^	3.4363	0.0052	0.0144	−0.0023	0.0029	0.3814	0.0308
		C21–C27 ^iv^	3.4015	0.0053	0.0151	−0.0023	0.0031	0.1980	0.0235
		C27–C21 ^iv^	3.4015	0.0053	0.0151	−0.0023	0.0031	0.1980	0.0235
		C25–C33 ^iv^	3.4363	0.0052	0.0144	−0.0023	0.0029	0.3814	0.0308
		C29–C23 ^iv^	3.4052	0.0057	0.0149	−0.0024	0.0031	0.8305	0.0300

Symbols: ρ(r)—electron density at the bond critical point (BCP); ∇^2^(r)—Laplacian electron density; V(r)—potential energy density; G(r)—kinetic energy; ε(r)—ellipticity of the electron density; d [Å]—the difference between the length of the bond path and the distance between the atoms linked by this bond.

**Table 5:** Decomposition of the interaction energy for stacking acridine molecules in the complexes of fenamic acids with acridine in kcal/mol.

	E_elect_	E_Pauli_	E_steric_	E_orb_	E_dysp_	E_total_	E_elect_
Fenamic acid^…^acridine	−10.51	21.97	11.46	−4.34	−18.54	−11.42	−10.51
**1**	−6.19	12.46	6.27	−2.45	−13.39	−9.57	−6.19
**2**_stack1	−9.08	18.62	9.54	−3.73	−17.47	−11.65	−9.08
**2**_stack2	−4.63	9.64	5.01	−1.81	−9.67	−6.47	−4.63
**3**_stack1	−4.02	8.33	4.31	−1.66	−9.26	−6.61	−4.02
**3**_stack2	−7.97	16.47	8.5	−3.37	−16.91	−11.77	−7.97

## Data Availability

Structural data are available in Cambridge Structural Database with deposition numbers: 2081569-2081571. Calculation data can be obtained from the authors upon request.

## Supplementary Materials

The following are available online, Table S1: Geometry of C–H∙∙∙π interactions in **2**.

## Sample Availability

Samples of the compounds are available from the authors.

## References

[1] Khansari P.S., Halliwell R.F. (2009). Evidence for neuroprotection by the fenamate NSAID, mefenamic acid. Neurochem. Int..

[2] Lee S.H., Bahn J.H., Whitlock N.C., Baek S.J. (2010). Actvating transcription factor 2 (ATF2) controlstolfenamic acid–induced ATF3 expression via MAP kinase pathways. Oncogene.

[3] Steed J.W. (2013). The role of co-crystals in pharmaceutical design. Trends Pharmacol. Sci..

[4] Schultheiss N., Newman A. (2009). Pharmaceutical Cocrystals and Their Physicochemical Poperties. Cryst. Growth Des..

[5] Jerzykiewicz L., Sroka A., Majerz I. (2016). The Crystal Structure and Behavior of Fenamic Acid-Acridine Complex under High Pressure. J. Pharm Sci..

[6] Bader R.F.W. (1990). Atoms in Molecules: A Quantum Theory.

[7] Johnson E.R., Keinan S., Mori-Sanchez P., Contreras-García J., Cohen A.J., Yang W. (2010). Revealing Noncovalent Interactions. J. Am. Chem. Soc..

[8] Alonso M., Woller T., Martin-Martinez F.J., Contreras-García J., Geerlings P., De Proft F. (2014). Understanding the Fundamental Role of π/π, σ/σ, and σ/π Dispersion Interactions in Shaping Carbon-Based Materials. Chem. Eur. J..

[9] Bone R.G.A., Bader R.F. (1996). Identifying and Analyzing Intermolecular Bonding Interactions in van der Waals Molecules. J. Phys. Chem..

[10] Popelier P.L.A. (1998). Characterization of a Dihydrogen Bond on the Basis of the Electron Density. J. Phys. Chem..

[11] Espinosa E., Molins E., Lecomte C. (1998). Hydrogen bond strengths revealed by topological analyses of experimentally observed electron densities. Chem. Phys. Lett..

[12] Espinosa E., Alkorta I., Rozas I., Elguero J., Molins E. (2001). About the evaluation of the local kinetic, potential and total energy densities in closed-shell interactions. Chem. Phys. Lett..

[13] Altun A., Neese F., Bistoni G. (2019). The Effect of Electron Correlation on Intermolecular Interactions: A Pair Natual Orbitals Coupled Cluster Based Local Energy Decomposition Study. J. Chem. Theory Comput..

[14] Majerz I., Gutmann M.J. (2011). Mechanism of proton transfer in the strong OHN intermolecular hydrogen bond. RSC Adv..

[15] Zhou T., Li F., Fan Y., Song W., Mu X., Zhang H., Wang Y. (2009). Hydrogen-bonded dimer stacking induced emission of aminobenzoic acid compounds. Chem. Commun..

[16] López-Mejías V., Kampf J.W., Matzger A.J. (2012). Nonamorphism in Flufenamic Acid and a New Record for a Polymorphic Compound with Solved Structures. J. Am. Chem. Soc..

[17] SeethaLekshmi S., Guru Row T.N. (2012). Conformational Polymorphism in a Non-steroidal Anti-inflammatory Drug, Mefenamic Acid. Cryst. Growth Des..

[18] López-Mejías V., Kampf J.W., Matzger A.J. (2009). Polymer-induced heteronucleation of tolfenamic acid: Structural investigation of a pentamorph. J. Am. Chem. Soc..

[19] Case D.H., Srirambhatla V.K., Guo R., Watson R.E., Price L.S., Polyzois H., Cockcroft J.K., Florence A.J., Tocher D.A., Price S.L. (2018). Successful Computationally Directed Templating of Metastable Pharmaceutical Polymorphs. Cryst. Growth Des..

[20] Zhikol O.A., Shishkin O.V., Lyssenko K.A., Leszczynski J. (2005). Electron density distribution in stacked benzene dimers: A new approach towards the estimation of stacking interaction energies. J. Chem. Phys..

[21] Wheeler S.E., Bloom J.W.G. (2014). Toward a More Complete Understanding of Noncovalent Interactions Involving Aromatic Rings. J. Phys. Chem. A.

[22] Kim K.S., Tarakeshwar P., Lee J.Y. (2000). Molecular Clusters of π-Systems: Theoretical Studies of Structures, Spectra, and Origin of Interaction Energies. Chem. Rev..

[23] Główka M.L., Martynowski D., Kozłowska K. (1999). Stacking of six-membered aromatic rings in crystals. J. Mol. Struct..

[24] Bora P., Saikia B., Sarma B. (2018). Regulation of π^…^π Stacking Interactions in Small Molecule Cocrystals and/or Salts for Physiochemical Property Modulation. Cryst. Growth Des..

[25] Morokuma K.J. (1971). Molecular Orbital Studies of Hydrogen Bonds. III. C=O^…^H–O Hydrogen Bond in H_2_CO^…^H_2_O and H_2_CO^…^2H_2_O. Chem. Phys..

[26] Ziegler T., Rauk A. (1997). On the calculation of bonding energies by the Hartree Fock Slater method. Theor. Chim. Acta.

[27] Herges R., Geuenich D. (2001). Delocalization of Electrons in Molecules. J. Phys. Chem. A.

[28] Sheldrick G.M. (2015). SHELXT—Integrated space-group and crystal-structure determination. Acta Cryst..

[29] Sheldrick G.M. (2015). Crystal structure refinement with SHELXL. Acta Cryst..

[30] Brandenburg K. (2014). DIAMOND.

[31] Frisch M.J., Trucks G.W., Schlegel H.B., Scuseria G.E., Robb M.A., Cheeseman J.R., Scalmani G., Barone V., Petersson G.A., Nakatsuji H. (2016). Gaussian 16, Revision A. 03.

[32] Becke A.D. (1993). Density-functional thermochemistry. III. The role of exact exchange. J. Chem. Phys..

[33] Lee C., Yang W., Parr R.G. (1988). Development of the Colle-Salvetti correlation-energy formula into a functional of the electron density. Phys. Rev. B.

[34] Keith T.A. (2019). AIMALL.

[35] Contreras-García J., Johnson E.R., Keinan S., Chaudret R., Piquemal J.-P., Beratan D.N., Yang W. (2011). NCIPLOT: A Program for Plotting Noncovalent Interaction Regions. J. Chem. Theory Comput..

[36] te Velde G., Bickelhaupt F.M., Baerends E.J., Fonseca Guerra C., van Gisbergen S.J.A., Snijders J.G., Ziegler T. (2001). Chemistry with ADF. J. Comput. Chem..

