# Supplementary material for: The Crystal Structure and Intermolecular Interactions in Fenamic Acids–Acridine Complexes
Source: Molecules. 2021 May 16;26(10):2956. doi: 10.3390/molecules26102956 (PMC8156396; doi:10.3390/molecules26102956)
Supplement: Supplementary file 1 [file molecules-26-02956-s001.zip › molecules-1200226-supplementary.pdf]

Supplementary Material

Table S1 Geometry of C–H···π interactions in **2**.

C–H···π	H···Cg (Å)	C···Cg (Å)	<(C–H···Cg) (°)
C14–H14a···Cg2^{i}	2.90	3.80	152
C15–H15b···Cg1^{ii}	2.91	3.69	137
C30–H30···Cg1^{iii}	2.92	3.72	143

Symmetry code: i = -x, -y, -z; ii = -1+x, y, z; iii = -x+1, -y+1, -z+1

Cg1 (C2/C3/C4/C5/C6/C7), Cg2 [C8/C9/C10/C11/C12/C13]
